# The Integrated Behavioural Model for Water, Sanitation, and Hygiene: a systematic review of behavioural models and a framework for designing and evaluating behaviour change interventions in infrastructure-restricted settings

**DOI:** 10.1186/1471-2458-13-1015

**Published:** 2013-10-26

**Authors:** Robert Dreibelbis, Peter J Winch, Elli Leontsini, Kristyna RS Hulland, Pavani K Ram, Leanne Unicomb, Stephen P Luby

**Affiliations:** 1 Social and Behavioural Interventions Program, Department of International Health, Johns Hopkins Bloomberg School of Public Health, Baltimore, MD, USA; 2 Department of Social and Preventive Medicine, School of Public Health and Health Professions, University at Buffalo, Buffalo, NY, USA; 3 Water, Sanitation, and Hygiene Research Group, Centre for Communicable Diseases, icddr,b, Dhaka, Bangladesh; 4 Woods Institute of the Environment, Stanford University, Stanford, CA 94305, USA

**Keywords:** Behavioural theory, Behaviour change, Water, Sanitation, Hygiene, Technology, Systematic review

## Abstract

**Background:**

Promotion and provision of low-cost technologies that enable improved water, sanitation, and hygiene (WASH) practices are seen as viable solutions for reducing high rates of morbidity and mortality due to enteric illnesses in low-income countries. A number of theoretical models, explanatory frameworks, and decision-making models have emerged which attempt to guide behaviour change interventions related to WASH. The design and evaluation of such interventions would benefit from a synthesis of this body of theory informing WASH behaviour change and maintenance.

**Methods:**

We completed a systematic review of existing models and frameworks through a search of related articles available in PubMed and in the grey literature. Information on the organization of behavioural determinants was extracted from the references that fulfilled the selection criteria and synthesized. Results from this synthesis were combined with other relevant literature, and from feedback through concurrent formative and pilot research conducted in the context of two cluster-randomized trials on the efficacy of WASH behaviour change interventions to inform the development of a framework to guide the development and evaluation of WASH interventions: the Integrated Behavioural Model for Water, Sanitation, and Hygiene (IBM-WASH).

**Results:**

We identified 15 WASH-specific theoretical models, behaviour change frameworks, or programmatic models, of which 9 addressed our review questions. Existing models under-represented the potential role of technology in influencing behavioural outcomes, focused on individual-level behavioural determinants, and had largely ignored the role of the physical and natural environment. IBM-WASH attempts to correct this by acknowledging three dimensions (Contextual Factors, Psychosocial Factors, and Technology Factors) that operate on five-levels (structural, community, household, individual, and habitual).

**Conclusions:**

A number of WASH-specific models and frameworks exist, yet with some limitations. The IBM-WASH model aims to provide both a conceptual and practical tool for improving our understanding and evaluation of the multi-level multi-dimensional factors that influence water, sanitation, and hygiene practices in infrastructure-constrained settings. We outline future applications of our proposed model as well as future research priorities needed to advance our understanding of the sustained adoption of water, sanitation, and hygiene technologies and practices.

## Background

The provision or promotion of low-cost water, sanitation, and hygiene (WASH) technologies at the individual, household, or community-level combined with hygiene promotion [1,2] is a key strategy for reducing diarrhoeal diseases in resource poor settings. Examples of these household-level technologies include handwashing stations to encourage handwashing with soap [3]; household-based water treatment with filters or chemical additives; chlorine dispensers for point-of-collection treatment of water from wells or standpipes [4,5]; and improved latrines [6]. A recent commission for *The Lancet* outlined how these and other “frugal” technologies – low-cost technologies that meet the specific needs of low-income countries – can make a significant contribution to global health [7].

In order for these interventions to result in meaningful improvements in population health, behaviours and technologies must be adopted and maintained over time at scale, but evidence of sustained adoption of new practices is mixed. While some studies have reported significant increases in behavioural outcomes [8-13], others have demonstrated an attenuation of initially improved practices and health impact [14-16]. These limitations to sustained adoption may reflect, in part, our still-developing understanding of the factors that influence WASH behaviour change and adoption of improved practices.

A number of researchers have identified factors – which we refer to as behavioural determinants – that influence the adoption of WASH technologies and the continuation of improved practices, and organized these determinants into theoretical frameworks or models. Use of behavioural theories or frameworks in the design and implementation of behaviour change interventions can result in improved behavioural outcomes [17-21]. After many years of WASH-related research and programmes, there are now a number of such models, some broad in scope and others specific to a single behaviour or social or environmental setting. While each has its merits, the profusion of these models makes it difficult to summarize findings across settings and outcomes and to compare determinants across different behaviours. Despite this wide range of theories and models available for use, their use in the design and assessment of WASH-related behaviour change activities is rare. For example, a systematic review of point-of-use water treatment interventions by Fiebelkorn et al. found that only seven of 26 published studies referenced any behavioural theory in intervention design and evaluation, and less than 2% of all published articles on point-of-use water treatment interventions reported on behavioural determinants [22].

In this paper, we outline a comprehensive framework for examining the behavioural determinants of WASH practices in order to inform intervention development and structure scholarly discussion on WASH behaviour change and maintenance.

## Methods

We conducted a systematic review of articles available in PubMed, identified through a combination of search terms associated with water, sanitation, and hygiene practices, with terms related to conceptual frameworks and models, and with names of key behaviour change theories and popular determinants referenced in existing water and sanitation research (see Table 1). No date restrictions were placed on our search.

**Table 1:** Search terms included in our systematic review

Terms related to behaviour change models and constructs	Health belief model OR social learning theory OR social cognitive theory OR conceptual model OR theory of reasoned action OR theory of planned behavior OR stages of change OR prochaska OR self-efficacy OR disgust OR shame OR psychological determinants OR behavioural determinants OR decision making
AND	
Terms related to WASH	Water OR soap OR handwashing OR latrine OR sanitation OR chlorine OR filter

Full citation information, including title, abstract, publication date, and journal name, was reviewed for all articles identified in the search. Articles that potentially included a behaviour change model or explanatory framework related to water, sanitation, and hygiene, were identified for full-text retrieval. From the grey literature, we identified documents that described conceptual models of behaviour change frameworks used by key global health organisations, such as Water and Sanitation Program (WSP) of the World Bank and the United States Agency for International Development (USAID). This included a review of WASH behaviour change approaches published by the Water Supply and Sanitation Collaborative Council [2]. Three criteria were used to further screen articles potentially employing behaviour change frameworks: 1) the framework addressed factors affecting WASH behaviours at one or more levels of aggregation (individual, household, community, etc.), 2) the framework drew, either implicitly or explicitly, from existing behavioural theory or presented a new theory/framework to summarize these factors, and 3) the framework related to WASH behaviours practiced in a community or domestic setting, rather than an institution (hospital, clinic) or private sector employer (restaurant, food services). Full texts were reviewed and information on behavioural models extracted. Both published and grey literature documents that did not present an explicit behaviour change model or framework but described itemized, specific behavioural determinants related to water, sanitation, and hygiene were excluded from our systematic review; however, relevant information on specific behavioural determinants was used to inform the development and elaboration of our emergent framework.

Findings from our review informed the development of our initial comprehensive behaviour change framework which guided technology selection and hygiene promotion for on-going formative and pilot research on the intervention content of two large-scale cluster randomized trials to be conducted by the International Centre for Diarrhoeal Disease Research, Bangladesh (icddr,b)^a^. Feedback from these formative and pilot projects was reviewed iteratively with our emergent model, and led to the subsequent organisation of the initial framework into three Dimensions (contextual, psychosocial, technological) and five aggregate Levels (behavioural, individual, interpersonal/household, communal, societal).

The resulting multi-level behaviour change framework – the Integrated Behavioural Model for Water, Sanitation, and Hygiene (IBM – WASH) was presented at the 2011 Oklahoma University International WaTER Conference, and at participatory workshops and lectures at iccdr,b in Dhaka and Johns Hopkins Bloomberg School of Public Health in Baltimore, Maryland. Feedback from these presentations was noted and incorporated into the framework. The full description of the model is the focus of this publication. IBM – WASH subsequently served to develop a codebook for the analysis and interpretation of qualitative findings from the concurrent formative and pilot research on handwashing, point-of-collection or point-of-use water treatment, and sanitation technologies and behaviours (data not shown). Results from one such analysis are presented in Hulland et al. [23].

## Results

### Systematic review of existing behaviour change models and frameworks in WASH

Our initial search criteria identified a total of 930 references. Citations that did not present an explicit or implicit conceptual model or behavioural framework were excluded, resulting in a total of 49 articles for full-text retrieval. Of those, a total of 15 references with behaviour change or decision-making models were identified that met inclusion criteria and either presented an explicit conceptual model or behavioural framework or enough information on specific behaviour determinants to recreate an implicit model -12 from the published and three from the grey literature. Three of these references focused on food handling [24], hand hygiene [25], and water conservation [26] in high-income countries and were subsequently excluded from our synthesis. A model on water source management and regional governance in Bangladesh by Hurlimann et al. [27] met our inclusion criteria, yet the scope of the presented framework limited the ability to inform our main review questions. This left a total of 10 references corresponding to 8 models or frameworks identified through our systematic review. Table 2 presents an overview of the included models.

**Table 2:** Theoretical models of WASH and WASH-related behaviours included in the systematic review

**Citation**	**Behaviour or outcome of**	**Included determinants**
	**focus**	
Environmental Health Project et al. 2004 [1]	Diarrheal prevention	Access to hardware: water supply systems, improved sanitation, household technologies
		Hygiene promotion: communication, social mobilization, community participation, social marketing, advocacy
		Enabling environment: policy improvement, institutional strengthening, community organization, financing, partnerships
Rainey and Harding, 2005 [28]	Household water treatment (SODIS)	Application of the Health Belief Model, including:
		Individual perceptions: perceived severity and perceived susceptibility to disease (diarrhoea)
		Modifying factors: demographic variables, socio-economic variables, structural variables; perceived threat of disease; cues to action
		Likelihood of Action: perceived benefits of taking action minus perceived barriers, perceived efficacy of action and ability to complete it, likelihood of taking action
Jenkins and Scott, 2007 [32]	Sanitation	Preference (motivation): dissatisfaction with current practices, awareness of options
		Intention: priority of change among competing goals, absence of permanent constraints to acquiring sanitation
		Choice: absence of temporary constraints to acquiring sanitation
Curtis et al. 2009 (elaborated in Curtis et al. 2011) [34,35]	Handwashing with soap	Planning: teaching children manners
		Motivation: disgust, norms, conform, nurture
		Habit: train children, tips to train oneself
		Social norms
		Physical facilities: cues, costs
		Biological signs of contamination
Devine, 2009 / Coombes and Devine, 2010 [39,67]	Handwashing (FOAM) and Sanitation (SaniFOAM)	Opportunity: access / availability, product attributes, social norms (FOAM), sanction/enforcement (SaniFOAM)
		Ability: knowledge, social support (FOAM), skills and self-efficacy, roles and decisions, affordability (SaniFOAM)
		Motivations: beliefs and attitudes, outcome expectations, threat, intention (FOAM), values, emotional/physical/social drivers competing priorities, willingness-to-pay (SaniFOAM)
Figueroa and Kincaid, 2010 [37]	Household water treatment and storage	Individual: knowledge / skills, attitudes, perceived risk and severity, subjective norms, self-image, emotional response, self-efficacy, empathy & trust, social influence, personal advocacy
		Household: time allocation, family support, resources, decision making
		Community: value for water quality, leadership, action, resources, cohesion
		Environmental/context: burden of disease, WASH technologies, community infrastructure, socio-demographic infrastructure, income inequality
Wood et al. 2011 [31]	Household water treatment (filters)	Awareness: Perceived need, awareness of products, assess value of products and relevance to lives
		Action: trial / initial use, sustained use
		Maintenance: purchase, sustained use
Mosler, 2012 [36]	WASH practices (general)	Risk factors: perceived vulnerability, perceived severity, factual knowledge
		Attitude factors: Instrumental beliefs, affective beliefs
		Normative Factors: descriptive, injunctive, and personal norm
		Ability Factors: Action knowledge, self-efficacy, maintenance efficacy, recovery efficacy
		Self-Regulation Factors: action control / planning, coping planning, remembering, commitment

Rainey and Harding [28] examined the adoption and use of solar disinfection for water treatment in Nepal using a modified version of the Health Belief Model [29,30], in which the relationship between individual perceptions and behavioural outcomes are linked by modifying factors, such as: perceived threat, individual socio-demographics, and cues to action. Two models focus on the decision making process related to the adoption and/or sustained use of specific WASH enabling technologies, one on water treatment technologies in Malawi [31] and the other on household sanitation in Benin [32]. Both models either implicitly or explicitly draw from Prochaska’s Transtheoretical (Stages of Change) Model [33].

Curtis et al. [34] present a conceptual model of handwashing behaviours based on an 11-country review of formative research findings in support of national-level handwashing promotion programs. The authors propose that three separate but interacting mental processes – planning, motivations, and habits - shape behaviour. Each of these three is, in turn, shaped by environmental determinants that include the biological, the social, and the physical. The specific determinants are further elaborated in a review paper [35].

The RANAS model by Mosler [36] divides behavioural determinants into five primary factor “blocks” – risk factors, attitudinal factors, normative factors, ability factors, and self-regulation factors. Of the models identified, the RANAS model is one of the few that is intended to be applicable across multiple WASH practices and interventions. The RANAS model associates specific intervention strategies with each of the identified factor blocks – information interventions with risk factors; persuasive interventions with attitudinal factors; infrastructural and ability interventions with ability factors.

Our review also included three primarily programmatic frameworks. Figueroa and Kincaid [37] developed a model of communication for water treatment and safe storage practices. According to their model, interventions influence behavioural outcomes via a set of multi-level intermediary outcomes. Individual-level outcomes included in the framework are further divided into cognitive elements, emotional factors, and social interactions. Household factors include time allocation, household-decision making practices, and household income. Community-level factors include community action and resources, community cohesion, and community leadership.

FOAM (for handwashing) and SaniFOAM (for sanitation behaviours) are behavioural frameworks developed by the World Bank’s Water and Sanitation Program [38,39]. They organize behavioural determinants into three main domains – *Opportunity* to improve a particular behaviour, *Ability* to change behaviour, and *Motivation* to change behaviour. Product attributes - such as comfort, convenience, and smell - are considered opportunity determinants. Willingness-to-pay, particularly for sanitation improvements, is viewed as a critical component of motivational determinants.

The Hygiene Improvement Framework, developed by the Environmental Health Project in partnership with USAID, UNICEF, and others, proposes a framework for combating diarrhoeal disease that consists of three main components: improving access to water and sanitation hardware, promoting hygiene, and strengthening the enabling environment [1].

### Discussion of systematic review findings

Each of the models identified in this review offers valuable theoretical and conceptual determinants that help us understand the nature of WASH behaviours and WASH behaviour change; however a number of other factors were not emphasized in the existing models. Characteristics of the type of water and sanitation technology (e.g. handwashing station, water treatment technology, latrine), including the cost and complexity of using it were under-represented in existing frameworks. Contextual factors – i.e.: gender, age, socio-economic status, household structure and availability of resources - were explicit in only a limited number of existing frameworks, such as the model proposed by Figueroa and Kinkaid; or selected contextual factors, such as roles in decision making, and affordability (FOAM/SaniFOAM), were grouped with other psychological or social determinants of behavioural outcomes.

Most existing models concentrated almost exclusively on individual-level factors that influence behavioural outcomes rather than utilized a broader ecological model approach that positions individual behaviours within a multi-level causal framework [40,41], a trend that is consistent across much of public health research [42]. Those frameworks that do incorporate a multi-level perspective do so only for psychological factors related to behaviour change [34,37]. While intervening at individual, community, *and* policy levels may be beyond the scope of many WASH interventions, understanding WASH behaviours within their spheres of influence is an important step in creating and sustaining behaviour change. In their application of the Health Belief Model to solar disinfection in Nepal, Rainey and Harding [28] discuss how structural factors, such as gender roles and involvement in agricultural production limit the time women have and are willing to commit to household water treatment. Characteristics of the physical environment such as chemical and microbiological composition of available sources of water, level of the water table, pattern of precipitation, population density, and available space were largely ignored.

Attributes or characteristics of WASH behaviours, such as the steps to be followed, and when and where the behaviour should be carried out in order to have a health impact [43] and factors affecting whether a behaviour becomes habitual [44] were rarely considered in existing frameworks. Habit may be particularly important in relation to WASH practices. Improved water, sanitation, and hygiene practices are not one-time behaviour changes – they require significant repetition across both time and space. The frameworks proposed by Jenkins and Scott [32] and Wood et al. [31] presented decision making models related to adoption of specific technologies, but only the model from Wood et al. explicitly addressed factors related to sustained and continued use and maintenance of technologies^b^.

### The Integrated Behavioural Model for Water, Sanitation, and Hygiene

The Integrated Behavioural Model for Water, Sanitation, and Hygiene (IBM-WASH) represents our synthesis of these existing behavioural models, our review of the evidence base for a number of other behavioural determinants not emphasized in those models, and feedback from concurrent formative and pilot research as mentioned in the Methods. IBM-WASH takes the form of a matrix, with three dimensions (columns) and five levels (rows), consistent with the matrices of ecological frameworks (See Table 3).

**Table 3:** The Integrated Behavioural Model for Water, Sanitation, and Hygiene (IBM-WASH)

**Levels**	**Contextual factors**	**Psychosocial factors**	**Technology factors**
**Societal/Structural**	Policy and regulations, climate and geography	Leadership/advocacy, cultural identity	Manufacturing, financing, and distribution of the product; current and past national policies and promotion of products
**Community**	Access to markets, access to resources, built and physical environment	Shared values, collective efficacy, social integration, stigma	Location, access, availability, individual vs. collective ownership/access, and maintenance of the product
**Interpersonal/Household**	Roles and responsibilities, household structure, division of labour, available space	Injunctive norms, descriptive norms, aspirations, shame, nurture	Sharing of access to product, modelling/demonstration of use of product
**Individual**	Wealth, age, education, gender, livelihoods/employment	Self-efficacy, knowledge, disgust, perceived threat	Perceived cost, value, convenience, and other strengths and weaknesses of the product
**Habitual**	Favourable environment for habit formation, opportunity for and barriers to repetition of behaviour	Existing water and sanitation habits, outcome expectations	Ease/Effectiveness of routine use of product

Our framework has three intersecting dimensions that influence WASH-behaviours: the contextual dimension, psychosocial dimension, and the technological dimension. The **Contextual Dimension** includes determinants related to the individual, setting, and/or environment that can influence behaviour change and adoption of new technologies. The **Psychosocial Dimension** comprises the behavioural, social, or psychological determinants that influence behavioural outcomes and technology adoption. The specific attributes of a technology, product, or device that influence its adoption and sustained use constitute the **Technological Dimension**. These three interacting dimensions reflect the concept of reciprocal determinism in Social Cognitive Theory, which describes mutual interactions between the individual, the behaviour, and the environment in which the behaviour is practiced [45].

For the purposes of our framework we have identified five aggregate levels generally analogous to levels that exist in multi-level models. The **Societal / Structural Level** of our framework refers to the broad organisational, institutional, or cultural factors that influence behaviours in each of our three dimensions. This includes factors such as laws, policies, climate, geography, geology, and manufacturing and commercial distribution of products. The **Community Level** includes the physical and social environment in which individuals are nested, as well as the formal and informal institutions that shape individual experiences. It is analogous to both the Institutional and the Community factors as proposed by McLeroy et al. [40]. The **Interpersonal / Household Level** represents interactions between individuals and the people they intimately associate with, including household members, close friends and neighbours. At this level, factors include roles and responsibilities in the household, household wealth, injunctive and descriptive norms, aspirations, shame, sharing access to a product, and behavioural modelling. The **Individual Level** includes sociodemographic factors – such as age and gender, individual cognitive factors, and attitudes toward the product, hardware, or behaviour. The final level of our model is the **Habitual Level**. This level, nested within the individual, reflects the fact that the opportunity and necessity for WASH-related behaviours are repeated over the course of the day, and the multiple processes or events that can result in the specific behavioural outcomes. Given emerging evidence on the importance of habit and automaticity in promoting and maintaining behaviour change, our framework also focuses on factors related to habit formation.^b^

### Dimensions of IBM-WASH: contextual factors, psychosocial factors, and technology factors

#### Contextual dimension

The contextual dimension represents the background characteristics of the setting, individual, or environment that are often beyond the scope of influence of program activities; however, they exert significant influence on the adoption of specific products or behaviours. These include access to markets and products, access to enabling resources (such as water for handwashing or water treatment), socioeconomic and demographic characteristics, characteristics of the household, and the built and natural environment. Many of the structural or societal determinants included in the contextual dimension are well summarized in the WASH Poverty Index [46], a multidimensional thematic tool for the integrated analysis of WASH and poverty linkages which approaches WASH from a policy and development perspective. Individual determinants included in the contextual dimension are similar to the “Background Characteristics” included in later iterations of the Health Belief Model [29,30].

There are only a few studies that have explicitly considered the relationship of these contextual factors with WASH behaviours. In a study of handwashing practices in Kenya, Schmidt et al. [47] found that both media exposure and media ownership were associated with increases in handwashing with soap, while constraints imposed by the natural environment, such as limited water and sanitation access, and individual socio-demographic characteristics (e.g. lower education) were associated with reduced rates of handwashing with soap among the most impoverished population groups. Contextual factors played a key role in household decision making regarding sanitation adoption in the decision-making framework proposed by Jenkins and Scott based on work in Ghana [32]. Physical constraints, limited credit, and availability of funds, as well as poor soil conditions prohibited households from moving to later decision making stages. Multiple contextual factors – including population mobility, gendered division of labour, and economic constraints – were identified as barriers to effective environmental sanitation and clean water provision in *colonias* along the Mexican-US border [48].

The context in which behaviour occurs is dynamic and changes throughout the day – children go to school, adults go to work, household members go to the market. The final level of the Contextual Dimension explicitly addresses these by identifying other opportunities or the lack of other opportunities to repeat and continue practicing an improved behaviour. Understanding handwashing behaviours among school children at home must be understood within the context of handwashing water, soap, and facilities available at schools. The benefits of drinking safe water at home will be limited by drinking unclean drinking water at the place of employment. There may be important variations in access to improved technologies and opportunities to practice improved behaviours within the same community setting. Understanding and recognizing these variations is an important part of developing a complete understanding of the contexts in which behaviours occur.

#### Psychosocial dimension

The psychosocial dimension of the IBM-WASH model consists of factors that are amenable to intervention activities. These are often the focus of behaviour change strategies. Psychosocial factors have been described by various names in models such as the Health Belief Model [30,49], the Theory of Reasoned Action and Theory of Planned Behaviour [50,51], and Social Cognitive Theory [45]. In operational frameworks, such as FOAM, psychosocial factors are often referred to as “behavioural determinants” [38,39]. For Figueroa and Kincaid, they are referred to as intermediate outcomes in the path towards behaviour change [37]. For Aunger et al., psychosocial factors are identified as the psychological determinants related to behaviours [52]. The factor blocks presented by Mosler [36] represent another way to organize the psychosocial determinants described in the IBM-WASH model.

Disgust has been one of the most widely researched psychosocial determinants related to WASH – particularly in relation to handwashing with soap and open defecation [53-56]. Experimental evidence from Australia and the United Kingdom has shown an association between disgust messages and handwashing behaviours [57,58]. In Community Led Total Sanitation (CLTS), elicitation of disgust at the community level is a key step in mobilising support for sanitation improvements [56]. Social norms and/or social desirability, and aspirations are also widely acknowledged to influence WASH practices as well as play a central role in Diffusion of Innovation Theory [6,55,59-61]. Nurture and motherhood / caretaking are important psychosocial factors related to hygiene and handwashing practices [62]. Knowledge and perceived threat of illness – particularly diarrhoeal disease – are often key components of behaviour change promotion strategies. While some studies have linked knowledge with improved hygiene practices in a domestic environment [63], the role of knowledge alone in motivating WASH behaviour in the domestic environment has been questioned by researchers. While beliefs, perceived behavioural control, and self-efficacy have been associated with improved WASH practices in a number of specific institutional settings [25,64], a general exploration of their role in domestic WASH has been largely absent from the existing literature. At higher levels of our framework, factors such as community cohesion and social integration have been found to influence the success of interventions [48,61]. We present an example of the psychosocial dimension of our multi-level framework applied to use of community-level chlorine dispensers in Table 4^c^.

**Table 4:** Application of the psychosocial dimension of the IBM-WASH framework to community-based chlorine dispensers

	**Psychosocial factors**	**Example for chlorine dispsenser**
**Societal/Structural**	Leadership/advocacy; cultural identity	• Political commitment and donor driven priorities.
		• Commitment and dedication of national government to promoting chlorination.
**Community**	Shared values, collective efficacy, social integration, stigma	• Community commitment to practice chlorination.
		• Local leadership.
		• Collective efficacy for supporting and maintaining water treatment practices.
**Interpersonal/Household**	Injunctive norms, descriptive norms, aspirations, shame	• Perceived prevalence of chlorination among local and broader social network.
		• Perception of the extent to which others in social network expect someone to adhere to chlorination practices.
		• Aspirations related to nurture/safe motherhood and maintaining a clean and healthy child.
**Individual**	Self-efficacy, knowledge, perceived threat, disgust	• Knowledge of the transmission of diarrheal disease and perceived threat of associated illness.
		• Disgust reaction to contaminated drinking water.
		• Self-efficacy of identifying supplies, taking necessary time, and completing necessary steps to maintain clean water.
**Habitual**	Existing habitual behaviours, outcome expectations	• Existing water treatment practices (boiling, traditional filters).
		• Expectation for chlorination (taste, colour, smell).

#### Technological dimension

All WASH practices – even simple handwashing with soap – require some type of physical product or technology component, and characteristics of this hardware can often have a strong influence on behavioural outcomes. First, the location of the technology required to carry out behaviour may facilitate or inhibit practice. Having soap or water at a convenient location for handwashing was associated with improved handwashing practices following faecal contact in rural Bangladesh [65]. The Water and Sanitation Program’s (WSP) Global Scaling Up Handwashing Project has identified the importance of enabling products in handwashing, linking handwashing technology and behaviour change [66]. In a study assessing handwashing station design, Devine found that physical characteristics of the handwashing station, including tap design, soap presentation, and container parameters, influenced acceptability and use [67]. Ease of use can influence technology preference and behaviour change. In a structured decision making study of various point-of-use water treatment technologies in rural Tanzania, research participants prioritized the ease and convenience of water treatment with liquid sodium hypochlorite solution over more effective yet less convenient alternative point-of-use technologies [68]. In addition to factors that influence the acceptability of a technology, physical attributes also determine what technologies are feasible in different settings. In a study testing sanitation facilities in urban slums in Uganda, investigators reported that in order for a latrine to be sustainable the technology must be durable, that materials must be available for construction and on-going maintenance, and that the system must be upgradeable [69]. Table 5 provides an example of the technology dimension of the IBM-WASH model applied to the same example of chlorine dispensers.

**Table 5:** Application of the technology dimension of the IBM-WASH framework to community-based chlorine dispensers

	**Technology factors**	**Example for chlorine dispensers**
**Societal/Structural**	Manufacturing, financing and distribution, current and past national policies and promotion of products	• Manufacturing capacity for chlorine (powder or liquid)
		• Distribution and delivery of supplies to refill and maintain chlorine dispensers
**Community**	Location, availability, individual vs. collective ownership/access and maintenance of the product	• Position of dispensers near community or private water points
		• Ownership and accountability for maintaining, refilling, and/or repairing dispensers
**Interpersonal/Household**	Sharing of access to product, modelling/demonstration of use of product	• Restrictions on access to chlorine dispensers
		• Dispensers placed in public/open location to allow for modelling and observation of the behaviour
**Individual**	Perceived cost, value, convenience and other strengths and weaknesses of the product	• Fees or payments for chlorine dispenser access
		• Negative reaction to chlorine smell in drinking water
		• Low perceived need
**Habitual**	Ease / Effectiveness of routine use of product	• High perceived effectiveness of chlorine
		• Convenient access at time of water collection, visible cues to action re: water treatment

While we have presented the three dimensions of our framework separately and in relation to specific behaviours, it is our intent that the contextual, psychosocial, and technological dimensions of any particular WASH behaviour be viewed collectively. The complete IBM-WASH model with all three dimensions and five levels is presented in Table 3. However, in order to demonstrate the framework’s complete application to a specific behaviour, we present an example of the IBM-WASH model applied to the use of potties for disposal of child faeces in Table 6. This application is based on formative research activities in Bangladesh. It is beyond the scope of this review, however, to present the data and analysis that generated this application.

**Table 6:** The full IBM-WASH framework applied to the use of child potties

**Levels**	**Contextual factors**	**Psychosocial factors**	**Technology factors**
**Societal/Structural**	Rainy and dry seasons and their effect on child defecation habits. Type of soil	Leadership / advocacy for use of child potties	Manufacturing capacity for child potties; national policies re: child defecation
**Community**	Access to latrines, sewers, potable water in the community	Shared values, collective efficacy for community-wide use of potties	Availability and distribution of child potties in the community
**Interpersonal/Household**	Household members and division of labour related to child-care and disposal of child faeces; condition of the latrine	Injunctive norms, descriptive norms for child potty use; responsibility for cleaning potty at household level	Sharing of access to product, modelling/demonstration of use of product
**Individual**	Wealth, education and employment of caretaker of child; age and developmental stage of child and their effect on potty use	Self-efficacy for potty training of child and correct use of potty; knowledge of diarrheal diseases; disgust and perceived threat related to child faeces in the household or courtyard	Strengths and weaknesses of child potties for end-users; adaptation of design to respond to consumer preferences
**Habitual**	Favourable environment for formation potty using habit, and regular emptying of potty; defecation away from home and its impact on habit formation	Existing habits for disposal of child faeces; outcome expectations: What is the expected outcome of consistent potty use by the child	Ease / Effectiveness of routine use of child potties, need for potty training; visible potty as cue to action for potty use

## Discussion

In a review on the role of theory in behavioural health interventions, Crosby and Noar [70] identify three aspects of theory and theory development that can be viewed as criteria against which specific behavioural theories can be judged: 1) Theories should be derived from practice, 2) Theories should transcend the individual level, and 3) Theories should be “accessible” to practitioners. It is our goal that development and application of IBM-WASH across a number of water, sanitation, and hygiene interventions results in both a theoretical and operational framework that fulfils these criteria.

### Theory derived from practice

Our framework emerged not only through a review of existing theories and theoretical models, but was iteratively adapted based on its application during formative research and intervention piloting in support of two large-scale cluster randomized trials in Bangladesh. For example, earlier versions were focused on individual-level psychosocial determinants with relatively little focus on other levels or the complex interactions between psychosocial determinants and context and technology. The technological component was initially based solely on individual-level determinants as well, and the application to technologies shared among multiple households – such as chlorine dispensers – informed the multi-level approach to each of the three dimensions of our framework. Initial iterations consisted of a traditional “boxes and arrows” approach to presenting behaviour change theories. As our framework expanded, we modified our presentation to a matrix format, focusing on relationships between and among determinants rather than causal pathways. Further feedback informed the inclusion of the habitual level. It is beyond the scope of this manuscript to provide details on each stage of the evolution of IBM-WASH, however this process was used to identify redundancies in behavioural determinants in our initial summary model, identify factors that required more or less elaboration, and inform the explicit use of a multi-level framework. Detailed results from several of the concurrent pilot projects include technologies for child faeces management [71], handwashing station designs and selection [23], and point-of-use water treatment technologies (manuscript in preparation).

One key application of the IBM-WASH model is to guide researchers and practitioners in acknowledging the various levels of influence that may shape behavioural-level outcomes, through the use of individual cells as a “checklist” in order to ensure that the full set of determinants for a specific behavioural intervention have been considered prior to implementing an intervention. Such a checklist can help identify areas in which additional qualitative data collection will help to understand the technological, psychosocial, and contextual dimensions that shape specific behavioural outcomes. In a similar manner, our framework can be applied to the development of data collection tools and instruments intended to better understand WASH-behaviours. The structure of our matrix easily translates to the development of qualitative codebooks and analysis plans for qualitative data (see Hulland et al. 2013 for an example).

### Transcending the individual-level

Structured as a multi-level model, the IBM-WASH facilitates the process of developing interventions that operate beyond the individual or household level. Interventions that operate at the structural level not only have the capacity to reach large sections of the population, but are also highly cost-effective [72].

### “Accessible” to practitioners

While specific determinants and their relative contribution to behaviour change and habit formation may differ between behaviours, technologies, or populations, the IBM-WASH model is intended to be simple, adaptable, and accessible to both practitioners and researchers. Materials used for presenting and training researchers on the IBM-WASH framework are included as an on-line supplemental file (Additional file 1). The framework can help guide monitoring and evaluation strategies for both large- or small-scale WASH programs by identifying intermediate outcomes linking enabling technologies and hygiene promotion to specific behavioural outcomes. Tracking these intermediary outcomes provides a possible shorter-term outcome that can be used in on-going monitoring to evaluate the effectiveness of longer-term intervention activities or refine messaging and promotion strategies to influence key determinants more directly.

The intended accessibility of our framework to practitioners, however, underscores a number of specific research gaps in the WASH sector. The framework identifies a number of testable hypotheses applicable to WASH-related behavioural determinants, and rigorous approaches to the measurement of such determinants across all three domains of the framework are needed. Rigorous measurement of determinants and the application of measurement theory has been largely lacking within the WASH community. A handful of studies have focused on the validity and reliability of self-reported behavioural outcomes in both domestic and institutional contexts, and there is general agreement that alternative approaches to assessing behavioural outcomes are required [73-75]. There has been little to no attention given to the measurement of behaviour determinants within a domestic environment. The RANAS [36] model includes a suggestion for standardized questions and analyses that can be used to measure the behavioural factors included in the model; however, the measurement system proposed is based almost entirely on individual-level behavioural factors, nor does it address the complex inter-relationships among psychosocial, technological, and contextual determinants. Applications of the RANAS model have operationalized many of its determinants through single questions in a survey [76] and more information is required on the reliability and validity of the proposed measurement system. Studies have addressed the reliability and validity (or psychometric properties) of measures for specific behavioural determinants, including self-efficacy for hand washing in an institutional setting [63], disgust sensitivity [77] and habit strength [44]. Understanding the validity and reliability of scaled measures in low literacy populations – where there is a general lack of familiarity with both eliciting and providing scaled or Likert-type responses - would allow for a better understanding of their relative contribution to behaviour change and habit formation, and allow for comparisons among multiple behavioural outcomes.

We developed our model based on formative research for a select number of water, sanitation, and hygiene technologies, a potential limitation. These included various improved models of handwashing station design, technologies for cleaning child faeces, multiple point-of-use water treatment systems, and communal or household-level chlorination systems. Household water filters are one type of technology not included in the development of our model. Future research is needed in which the IBM-WASH framework is applied to and adapted for filter technologies. Regarding the Contextual Dimension of our framework, the durable nature of household filters associated with higher capital costs compared to chemical household water treatment systems may limit their appeal within lower income – and thus – higher need populations; while both the costs and visual appeal of filter technologies may appeal to the aspirational factors included in our Psychosocial Dimension. It is the Technology Dimension in which filters will likely differ the most from chemical-based treatment systems. Filters typically require fewer steps and less labour – thus easier to use - than chemical treatment options, which may facilitate adoption and habit formation. However, this perceived ease will be mediated by other strengths and weaknesses of the design including flow rate, wait time, and filtration effectiveness.

The determinants outlined in the IBM-WASH framework should be viewed as dynamic and inter-related. They may differ across place, setting, and time. There is general consensus, for example, on the fact that disgust may be a major determinant of WASH practices [34,35,52,55], yet there has been little attention to the ways in which the disgust response is shaped by factors related to the physical and natural environment. Exposure to open sewerage pits, industrial run-off, and crowded living conditions in an urban slum may result in populations with less sensitivity to disgust stimuli. Behaviours in schools, the street, and places of employment may be influenced by a different constellation of factors than in the home. At a minimum, behaviours outside of the home will be subject to a different set of contextual factors than in the domestic environment. In all cases, it is important to adapt the framework by defining the specific determinants operating at each level and dimension through formative and pilot research. The multiple levels of the IBM-WASH model ensure that any individual behavioural outcome must be understood within the larger societal and communal contexts in which it occurs. The potential complexities of these dynamic relationships among determinants are simplified by specifying a behaviour and/or a population of interest, a concept that is embedded in many programmatic behaviour change approaches and frameworks [38,39,78]. The focus of our framework is on behaviours and habit formation, and not explicitly on exposure reduction. It is assumed that improving WASH practices will reduce pathogen exposure, yet it is important to understand the determinants of these behaviours independent of their direct link with transmission pathways. Incorporating a dynamic view of the role of context in habit formation – specifically identifying opportunities and moments throughout the day within the community in which behaviours can or cannot be repeated – may guide practitioners in identifying other settings in which new behaviours and/or technologies are required. These other contexts within a community may also provide novel entry points for introducing new behaviours and technologies to a population.

## Conclusions

The success of interventions to improve WASH practices ultimately rests on the ability to foster and maintain behaviour change at the individual, household, community, and structural levels. A number of models have emerged in response to this role of interventions, yet existing frameworks suffer a number of limitations, such as a lack of focus on the contextual, psychosocial, and technology dimensions of WASH practices, or reliance on individual-level theories and outcomes. Moreover, theoretical models have been used rarely in the development and evaluation of WASH interventions [22]. The IBM-WASH framework provides a simple, adaptable tool for understanding WASH behaviours and habit formation that is informed by existing theoretical insights at multiple levels and dimensions. The development of our framework has also underscored a number of research gaps associated with WASH research and evaluation.

## Endnotes

^a^The first cluster-randomized trial measures the combined efficacy of a cholera vaccine along with promotion of handwashing and water chlorination at the point of collection in urban poor clusters, versus the efficacy of the cholera vaccine alone on diarrheal disease. The second cluster-randomized trial measures the single and combined efficacy of handwashing, point of use water chlorination, improved sanitation, and improved nutrition on diarrheal disease and child development.

^b^In a series of studies, social psychologist Bas Verplanken [44] has demonstrated that habit strength – including not only the frequency with which behaviours are practiced, but also the extent to which behaviours are automatic and beyond the conscious control of the individual – mediates the relationship between past and future behaviours. Data by Aunger et al. [52] found that pro-hygiene responses to survey questions related to habitual or automatic handwashing and disgust towards dirt and dirty hands, had a stronger association with observed hygiene practices, particularly handwashing following contact with stools, than responses related to motivations.

^c^Chlorine dispensers are usually positioned near pubic taps or other community water sources. Dispensers are designed to provide a specified concentration of liquid or powdered chlorine to collected drinking water. Unlike point-of-use water treatment, chlorine dispensers are used at the point of collection. Efficacy trials of chlorine dispensers are currently underway in Bangladesh and Kenya.

## Competing interests

The authors of this paper declare no competing interests.

## Authors' contributions

RD completed the systematic review, contributed to the synthesis and interpretation of results, contributed to the development of the final behavioural framework and was the primary author of the final manuscript. PW conceived of the work, developed the systematic review search criteria, contributed to the development of the final behavioural framework, and edited the manuscript. EL conceived of the work, contributed to the development of the final behavioural framework, provided support for multiple field trials and data collection activities used to inform the work, and edited the manuscript. KRSH contributed to the development of the final behavioural framework and drafted sections of the final manuscript. PKR, LU and SPL provided support for on-going field trials and data collection activities used to inform the work, drew attention to relevant concepts and studies in the WASH literature relevant to the work, and provided feedback on the manuscript. All authors read and approved the final manuscript.

## Pre-publication history

The pre-publication history for this paper can be accessed here:

http://www.biomedcentral.com/1471-2458/13/1015/prepub

## Supplementary Material

Additional file 1 IBM-WASH Intro Online Supplement. Click here for file
