# Supplementary material for: The Integrated Behavioural Model for Water, Sanitation, and Hygiene: a systematic review of behavioural models and a framework for designing and evaluating behaviour change interventions in infrastructure-restricted settings
Source: BMC Public Health. 2013 Oct 26;13:1015. doi: 10.1186/1471-2458-13-1015 (PMC4231350; doi:10.1186/1471-2458-13-1015)
Supplement: Additional file 1 — IBM-WASH Intro Online Supplement. [file 1471-2458-13-1015-S1.pdf]

IBM-WASH

Integrated Behavioral Model for Water, Sanitation and Hygiene

Reasons for creating IBM-WASH

Purposes of a model (1)

- The model described social and behavioral factors that affect adoption of water and sanitation behaviors such as:
  - Handwashing with soap
  - Chlorination of drinking water in the home
  - Construction, maintenance and use of latrines

Purposes of a model (2)

- Purposes of the model include:
  - Identification of factors to investigate in qualitative research that describes the reasons for WASH behaviors
  - Design of interventions to promote WASH behaviors
  - Design of data collection instruments to measure the effect of interventions on determinants of WASH behaviors

Why create yet another model?

- Other models are often specific to one behavior e.g. only for handwashing
- The technology (point of use water treatments, handwashing stations etc.) is rarely included in other models
  - Characteristics of the technology that favor or constrain adoption and use
  - People's responses to the technology
- Different levels of influence not acknowledged in many other models

IBM-WASH:
Organization of the framework

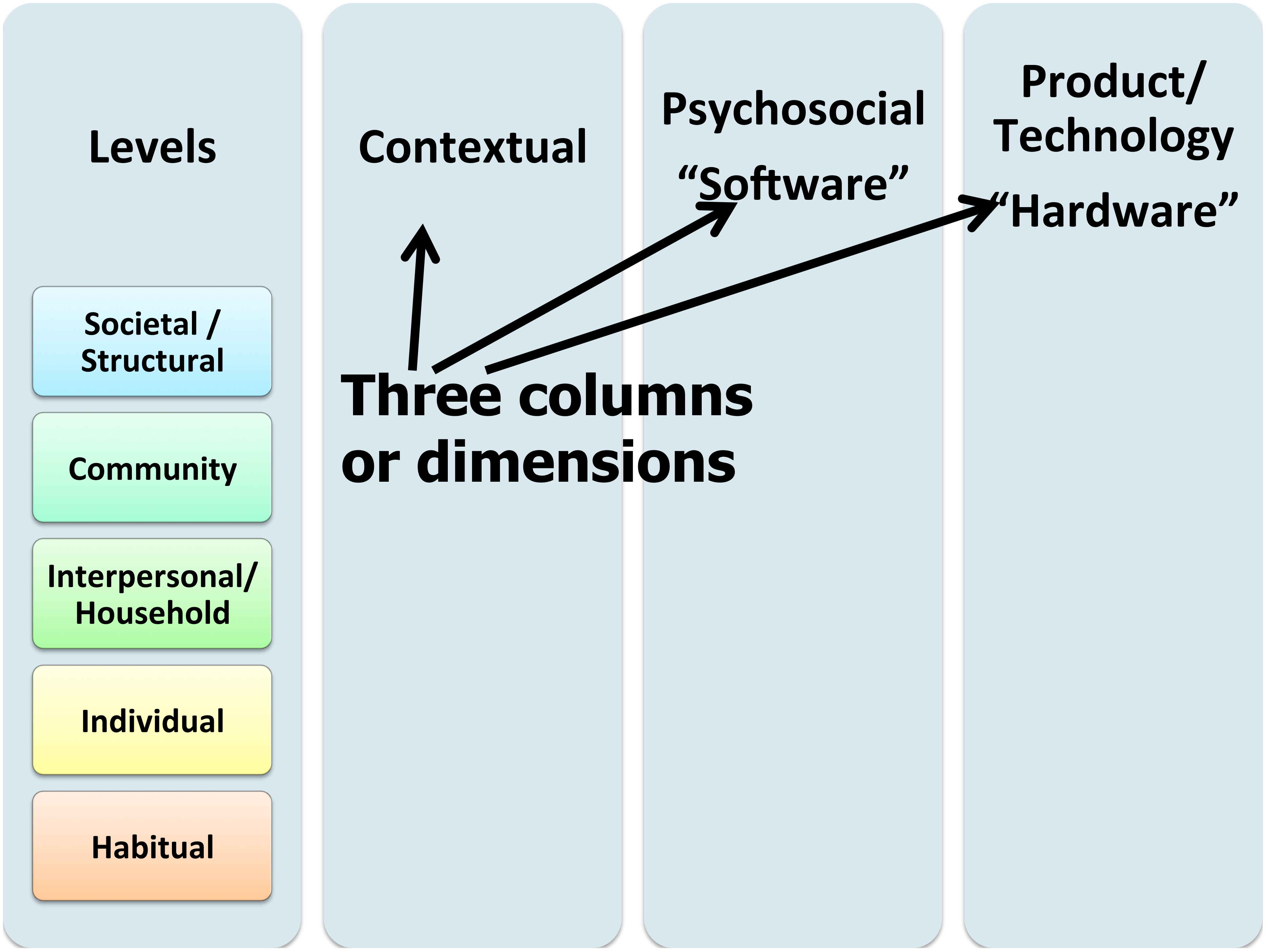

Levels
Contextual
Psychosocial
"Software"
Product/
Technology
"Hardware"
Societal /
Structural
Community
Interpersonal/
Household
Individual
Habitual
Three columns
or dimensions

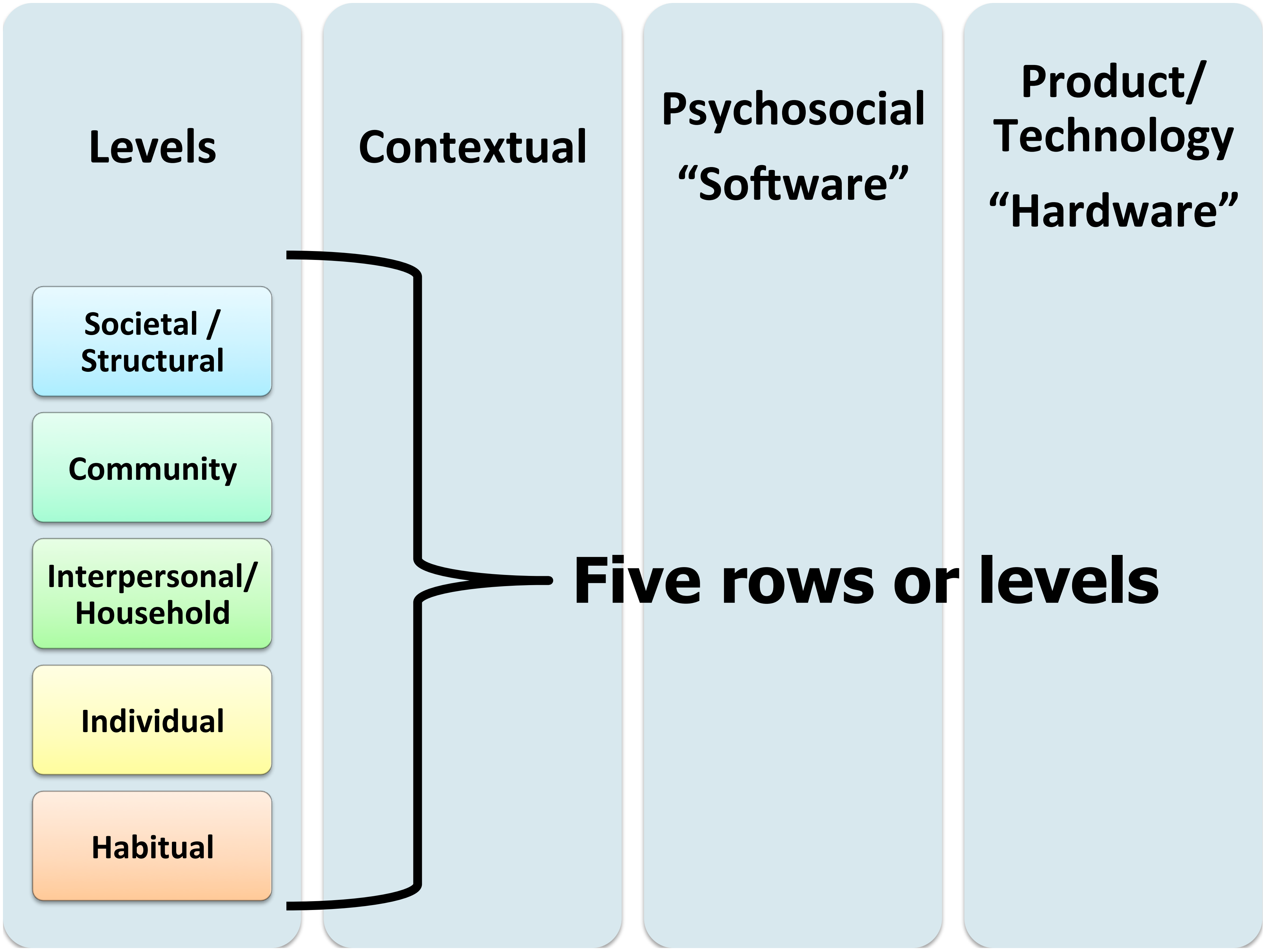
Levels
Contextual
Psychosocial
“Software”
Product/
Technology
“Hardware”
Societal /
Structural
Community
Interpersonal/
Household
Individual
Habitual
Five rows or levels

How we created IBM-WASH

Creating IBM-WASH

- We conducted a systematic review to find all existing models and frameworks of factors that affect adoption of WASH behaviors

Search strategy

- {Terms related to behavior change models and constructs}
- AND
- {Terms related to WASH}

Terms related to behavior change models and constructs

- health belief model OR social learning theory OR social cognitive theory OR conceptual model OR theory of reasoned action OR theory of planned behavior OR stages of change OR prochaska OR self-efficacy OR disgust OR shame OR psychological determinants OR behavioral determinants OR decision making

Terms related to WASH

- water OR soap OR handwashing OR latrine OR sanitation OR chlorine OR filter

- We left out (too many off-topic articles):
  - Defecation
  - Feces

Results of search

- Initial search: 931 references
- Reviewed abstracts, retained 49 references
- Identified 11 behavior change models or frameworks
  - Some explicit
  - Some implicit

10 references met 3 criteria

1. Presentation of factors affecting individual or household-level WASH behaviours
2. Draw from one or more existing behaviour change models to summarize these factors, and
3. Framework relates to WASH behaviours practiced in a community setting, rather than to an institution (hospital, clinic) or private sector employer (restaurant, food services)

Eight retained models

**Authors**	**WASH behaviors**	**Elements in model**
Rainey and Harding	Household water treatment	Health Belief Model: individual perceptions, perceived severity, perceived susceptiblity
Curtis et al.	Handwashing	Planning, motivations and habits
Figueroa & Kincaid	Handwashing	Indiv, HH & Comm levels<br>Cognitive elements, emotional factors, social interactions
FOAM, SaniFOAM	Handwashing, sanitation	Opportunity, ability, motivation
Wood et al.	Filters in Malawi	Prochaska: Stages of Change
Jenkins & Scott	Sanitation in Ghana	Prochaska: Stages of Change
Mosler	WASH practices (general)	Risk factors, Attitudinal factors, Normative Factors, Ability Factors, Self-regulation Factors
EHP et al.	Diarrhea prevention	Access to hardware, Hygiene Promotion, Enabling Environment

Training people to use IBM-WASH in their work

Training people to use IBM-WASH in their work

- The meaning of each row and column may not be intuitive to people
- When introducing the model to a new group of people, we have found it necessary to take them through an exercise of deciding where different factors should go: which row and which column
- What follows is such an exercise, for use in training people on IBM-WASH

IBM-WASH

Questions on classification of factors by dimension (column)

Levels	Contextual	Psychosocial "Software"	Product/ Technology "Hardware"
**Societal / Structural**	Policy and regulations, climate and geography	Leadership / advocacy, cultural identity	Manufacturing, financing and distribution of the product, current and past national policies and promotion of products
**Community**	Access to markets, access to resources, built and physical environment	Shared values, collective efficacy, social integration, stigma	Location, access, availability, individual vs. collective ownership/access and maintenance of the product
**Interpersonal/ Household**	Roles and responsibilities, household structure, division of labor	Injunctive norms, descriptive norms, aspirations, shame	Sharing of access to product, modeling/ demonstration of use of product
**Individual**	Wealth, age, education, gender, livelihoods/ employment	Self efficacy, knowledge, disgust, perceived threat	Strengths and weaknesses of technology for end-users, adaptation of design to respond to consumer preferences
**Habitual**	Favorable environment for habit formation, opportunity for and barriers to repetition of behavior	Existing water and sanitation habits, outcome expectations	Ease / Effectiveness of routine use of product

How do you decide which dimension/column?

- The factor cannot be addressed through an intervention e.g. high population density
  - Contextual
- The factor can be addressed by talking to people, changing their knowledge, skills, perceptions
  - Psychosocial
- The factor can be addressed by changing the technology e.g. changing volume of the reservoir, color of reservoir
  - Technology

Societal/structure level:
Contextual, Psychosocial or Technology?

- Handwashing station design is hard to manufacture, must be imported
- National law requiring that every restaurant have a sink with running water or a handwashing station for customers
- Commitment and dedication of national government to promoting handwashing
- National law – no tax on soap
- Three local companies exist that can manufacture handwashing stations

Societal/structure level:

Contextual, Psychosocial or Technology?

- Handwashing station design is hard to manufacture, must be imported (T)
- National law requiring that every restaurant have a sink with running water or a handwashing station for customers (C)
- Commitment and dedication of national government to promoting handwashing (P)
- National law – no tax on soap (C)
- Three local companies exist that can manufacture handwashing stations (C)

Community level:

Contextual, Psychosocial or Technology?

- Collective efficacy to control cholera epidemics through handwashing
- Water shortages, running water available only 3 hours per day
- Commitment by community organization to promoting handwashing
- Range of different housing types in community, different HWS designs needed
- Social norms for handwashing

Community level:

Contextual, Psychosocial or Technology?

- Collective efficacy to control cholera epidemics through handwashing (P)
- Water shortages, running water available only 3 hours per day (C)
- Commitment by community organization to promoting handwashing (P)
- Range of different housing types in community, different HWS designs needed for each housing type (C)
- Social norms for handwashing (P)

Interpersonal/household level:

Contextual, Psychosocial or Technology?

- Collective vs. individual responsibility for maintenance of the product and purchase of soap
- Shame if neighbor sees that you don’t wash your hands
- Large average household size, makes it difficult to share handwashing station
- Design of handwashing station means 3 people can use at one time

Interpersonal/household level:

Contextual, Psychosocial or Technology?

- Collective vs. individual responsibility for maintenance of the product and purchase of soap (P)
- Shame if neighbor sees that you don't wash your hands (P)
- Large average household size, makes it difficult to share handwashing station (C)
- Design of handwashing station means 3 people can use at one time (T)

Individual level:

Contextual, Psychosocial or Technology?

- People do not find the handwashing station attractive due to its color
- Self-efficacy to use handwashing station
- Handwashing station is expensive, due to more durable type of plastic used
- Perception that handwashing station is expensive
- Women work in garment factories, no time to refill HWS with water

Individual level:
Contextual, Psychosocial or Technology?

- People do not find the handwashing station attractive due to its color (T)
- Self-efficacy to use handwashing station (P)
- Handwashing station is expensive, due to more durable type of plastic used (T)
- Perception that handwashing station is expensive (P)
- Women work in garment factories, no time to refill HWS with water (C)

Habitual level:

Contextual, Psychosocial or Technology?

- Ease of using handwashing station
- Perceived benefits from using HWS
- No one is present during the day due to work, no opportunity to develop habit of using the handwashing station
- Existing handwashing habits

Habitual level:

Contextual, Psychosocial or Technology?

- Ease of using handwashing station (T)
- Perceived benefits from using HWS (P)
- No one is present during the day due to work, no opportunity to develop habit of using the handwashing station (C)
- Existing handwashing habits (P)

IBM-WASH

Questions on classification of factors by level (row)

Levels	Contextual	Psychosocial "Software"	Product/ Technology "Hardware"
Societal / Structural	Policy and regulations, climate and geography	Leadership / advocacy, cultural identity	Manufacturing, financing and distribution of the product, current and past national policies and promotion of products
Community	Access to markets, access to resources, built and physical environment	Shared values, collective efficacy, social integration, stigma	Location, access, availability, individual vs. collective ownership/access and maintenance of the product
Interpersonal/ Household	Roles and responsibilities, household structure, division of labor	Injunctive norms, descriptive norms, aspirations, shame	Sharing of access to product, modeling/ demonstration of use of product
Individual	Wealth, age, education, gender, livelihoods/ employment	Self efficacy, knowledge, disgust, perceived threat	Strengths and weaknesses of technology for end-users, adaptation of design to respond to consumer preferences
Habitual	Favorable environment for habit formation, opportunity for and barriers to repetition of behavior	Existing water and sanitation habits, outcome expectations	Ease / Effectiveness of routine use of product

How do you decide which level/row?

- To address the situation, you have to talk to policy makers/decision-makers in national, regional and district governments, owners of private enterprises, donor organizations: Changing laws and policies, increasing funding
  - Societal/structural
- To address the situation, you have to work with community groups/NGOs, local governments, women's groups: Community mobilization and empowerment, local capacity-building, CLTS (commitment to ending open defecation)
  - Community

How do you decide which level/row?

- To address the situation, you need to change how people are interacting with each other, how people divide up the work/responsibilities, how the household is organized: Household visits, counseling, negotiation of roles and responsibilities
  - Interpersonal/Household
- To address the situation, you need to increase individual knowledge, skills, perceptions
  - Individual

How do you decide which level/row? – Habitual

- To address the situation, you need to consider the behavioral recommendations, and the attributes of the recommended behaviors: Complexity of performing the behavior, how often does the behavior need to be practiced, are there visible benefits to practicing the behavior
- To address the situation, you need to ensure that the environment and the technology are conducive to habit formation: Cues to behavior, ease of repetition of the behavior, stable environment for performing the behavior

Contextual dimension:

Which level/row?

- Given local work routines (leaving early in morning to go to garment factory), it is difficult to use the handwashing station regularly
- Average household size is large, so many people need to share each handwashing station
- There is a national policy that all restaurants need a handwashing station (HWS)
- Community groups are very strong, and could help with promotion of HWS

Contextual dimension:
Which level/row?

- Given local work routines (leaving early in morning to go to garment factory), it is difficult to use the handwashing station regularly (Beh/Hab)
- Average household size is large, so many people need to share each handwashing station (Interp/ Household)
- There is a national policy that all restaurants need a handwashing station (Societal/structural)
- Community groups are very strong, and could help with promotion of HWS (Community)

Psychosocial dimension:

Which level/row?

- "If I don't use the handwashing station, my neighbors will think I am a bad person"
- After a participatory exercise facilitated by a local NGO, the entire community made a commitment to use handwashing stations
- HWS not used, no agreement within household on maintenance & purchase of soap
- If I use the handwashing station regularly, I will have less diarrhea and my hands will smell clean

Psychosocial dimension:

Which level/row?

- "If I don't use the handwashing station, my neighbors will think I am a bad person" (Interp.)
- After a participatory exercise facilitated by a local NGO, the entire community made a commitment to use handwashing stations (Comm)
- HWS not used, no agreement within household on maintenance & purchase of soap (Interpersonal/household)
- If I use the handwashing station regularly, I will have less diarrhea and my hands will smell clean (Behavioral/habitual)

Technology dimension:
Which level/row?

- HWS functions as a cue to practice handwashing
- HWS expensive because of the type of plastic
- HWS are easy to use
- People like HWS because they are attractive
- HWS are so large that they do not fit in the typical house, must be kept outside
- HWS can be sold at the local market
- Volume of HWS reservoir appropriate given number of users, and amount of water each used per day

Technology dimension:

Which level/row?

- HWS functions as a cue to practice handwashing (Habitual)
- HWS expensive because of type of plastic (Soc)
- HWS are easy to use (Habitual)
- People like HWS because they are attractive (Ind)
- HWS are so large that they do not fit in the typical house, must be kept outside (Inter/HH)
- HWS can be sold at the local market (Comm)
- Volume of HWS reservoir appropriate given number of users, and amount of water each used per day (Interpersonal/household)

Application to child potties

Levels	Technology "Hardware"
Societal / Structural	Manufacturing, financing and distribution of the product, current and past national policies and promotion of products
Community	Location, access, availability, individual vs. collective ownership/access and maintenance of the product
Interpersonal/ Household	Sharing of access to product, modeling/ demonstration of use of product
Individual	Strengths and weaknesses of technology for end-users, adaptation of design to respond to consumer preferences
Habitual	Ease / Effectiveness of routine use of product

Levels	Technology: Child potties
**Societal / Structural**	Manufacturing, financing and distribution of child potties, national policies and promotion of child potties
**Community**	Availability and distribution of child potties in the community
**Interpersonal/ Household**	Sharing of access to product, modeling/ demonstration of use of product
**Individual**	Strengths and weaknesses of child potties for end-users, adaptation of design to respond to consumer preferences
**Habitual**	Ease / Effectiveness of routine use of child potties, need for potty training. Visible potty as cue to action for potty use.

Levels	Psychosocial “Software”
Societal / Structural	Leadership / advocacy, cultural identity
Community	Shared values, collective efficacy, social integration, stigma
Interpersonal/ Household	Injunctive norms, descriptive norms, aspirations, shame
Individual	Self efficacy, knowledge, disgust, perceived threat
Habitual	Existing water and sanitation habits, outcome expectations

Levels	Psychosocial: Child potties
Societal / Structural	Leadership / advocacy for use of child potties
Community	Shared values, collective efficacy for community-wide use of potties
Interpersonal/ Household	Injunctive norms, descriptive norms for child potty use. Responsibility for cleaning potty at household level
Individual	Self efficacy for potty training of child and correct use of potty. Knowledge of diarrheal diseases. Disgust and perceived threat related to child feces in the household or courtyard
Habitual	Existing habits for disposal of child feces. Outcome expectations: What is the expected outcome of consistent potty use by the child?

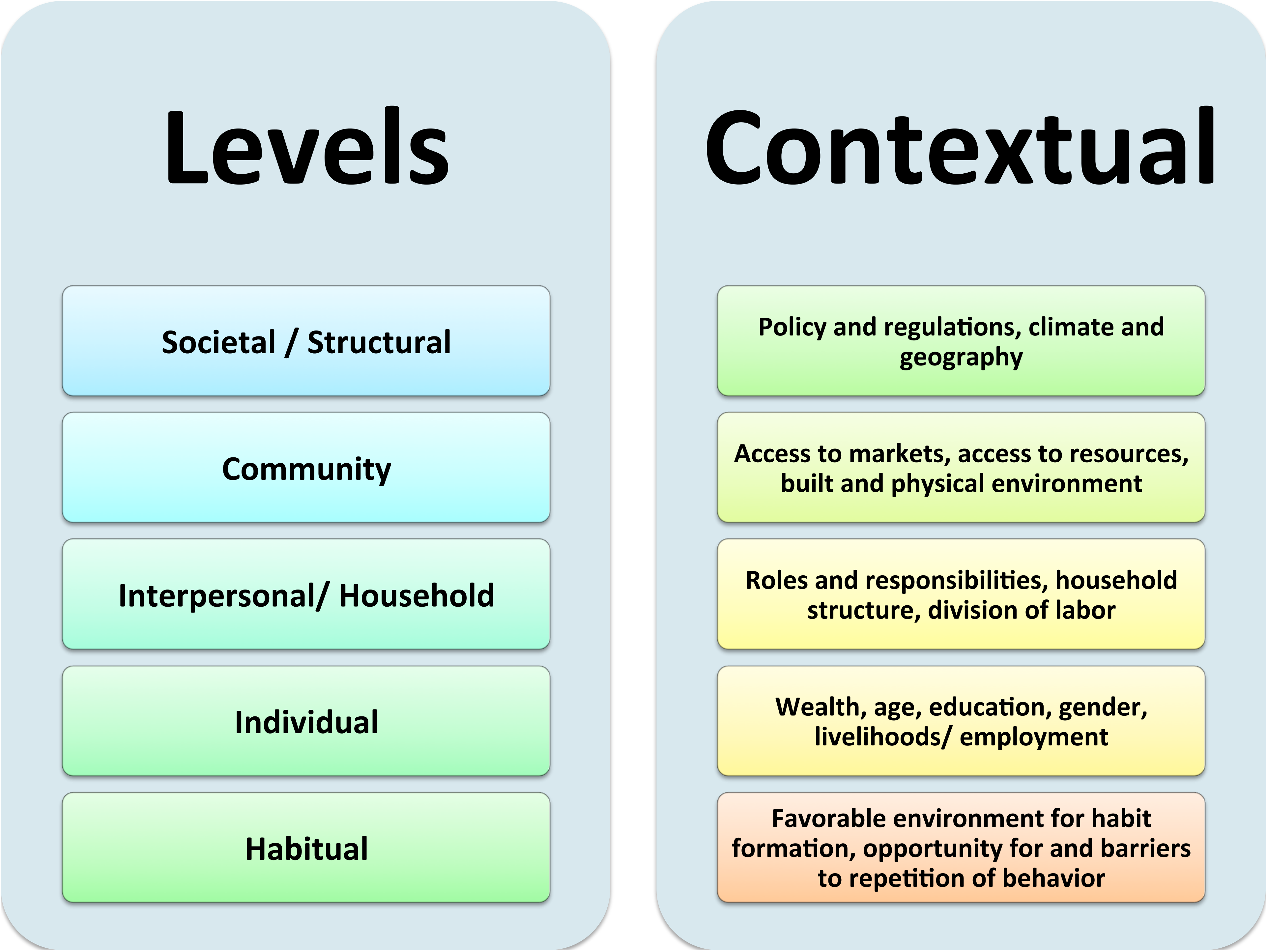
Levels
Contextual
Societal / Structural
Policy and regulations, climate and geography
Community
Access to markets, access to resources, built and physical environment
Interpersonal/ Household
Roles and responsibilities, household structure, division of labor
Individual
Wealth, age, education, gender, livelihoods/ employment
Habitual
Favorable environment for habit formation, opportunity for and barriers to repetition of behavior

Levels	Contextual: Child potties
**Societal / Structural**	Climate and geography: Rainy and dry seasons and their effect on child defecation habits. Type of soil
**Community**	Access to latrines, sewers, potable water in the community
**Interpersonal/ Household**	Household members and division of labor related to child care and disposal of child feces. Condition of the latrine
**Individual**	Wealth, education and employment of caretaker of child. Age and developmental stage of child and their effect on potty use
**Habitual**	Favorable environment for formation potty using habit, and regular emptying of potty
